# Hair Transplantation for Lichen Planopilaris: A Case Series of Five Patients

**DOI:** 10.3390/jcm14176199

**Published:** 2025-09-02

**Authors:** Katarzyna Osipowicz, Piotr Turkowski, Cezary Kowalewski, Janusz Pach, Piotr Regulski, Katarzyna Wozniak

**Affiliations:** 1 Department of Dermatology, Medical University of Warsaw, Nowogrodzka 59, 02-006 Warsaw, Poland; 2 OT CO Clinic, Bartycka 24B/U1, 00-716 Warsaw, Poland; 3 Department of Immunodermatology, National Medical Institute of the Ministry of the Interior and Administration, Wołoska str. 137, 02-507 Warsaw, Poland; 4 Laboratory of Digital Imaging and Virtual Reality, Department of Dental and Maxillofacial Radiology, Medical University of Warsaw, Binieckiego 6, 02-097 Warsaw, Poland

**Keywords:** alopecia, balding, hair loss, lichen planopilaris, surgery, treatment

## Abstract

**Background**: Although the history of treating alopecia with hair transplantation spans over 60 years, the literature providing evidence for the use of this method in patients with alopecia caused by lichen planopilaris (LPP) is extremely limited and primarily consists of isolated case reports. It has been suggested that these data may be subject to publication bias. Here, we describe a case series of five consecutive patients with LPP who underwent hair transplantation using the follicular unit extraction technique. **Methods**: Patient satisfaction was assessed twice (6 months and >12 months after transplantation), on a 5-point Likert scale, where 1 indicated no satisfaction at all and 5 indicated high satisfaction. **Results**: In the near follow-up, two patients reported strong dissatisfaction (40%), two patients were rather satisfied (40%), and one patient was very satisfied. In two cases, multiple surgeries were required to achieve a positive outcome. According to the physicians, all cases exhibited a positive therapeutic effect, which was documented with photographs. No complications were observed. In the extended follow-up, all patients declared satisfaction. **Conclusions**: Hair regrowth in patients with LPP is possible, despite the common belief that hair loss in LPP is irreversible.

## 1. Introduction

Assessing the progression of lichen planopilaris (LPP) and its treatment efficacy proves challenging, as no consistent markers are available, and treatment decisions are typically based on perceived severity. In 2010, the Lichen Planopilaris Activity Index was introduced to document the disease’s course and response to treatment, but this system primarily evaluates inflammatory symptoms and signs, making it a suitable tool for evaluating only chemical treatment [1].

Medical treatment standards for LPP are lacking. Current therapies for scarring alopecia typically involve topical and intralesional corticosteroids, finasteride or dutasteride, minoxidil, and hydroxychloroquine antibiotics to reduce inflammation and slow the progression of the disease, but their efficacy varies, and there are notable safety concerns [2]. However, medical therapy can only slow down the progression of the disease, leaving patients with noticeable hair loss, which negatively affects their psychological well-being [3]; therefore, surgical treatment of the esthetic consequences of this condition remains an important and unmet medical need [4].

Hair transplantation (HT) was initially described in 1959. Since then, it has evolved from mini/micrografts to single follicular unit transplantation through strip excision (follicular unit transplantation or FUT) and follicular unit extraction (FUE). Single follicular unit transfer is now considered the gold standard technique and has shown favorable outcomes in patients with androgenetic alopecia. However, for LPP, it remains a subject of controversy due to the scarcity of literature reporting results of this method in patients with this disease.

There are numerous unanswered questions regarding HT in patients with LPP, including the need for a quiescent disease phase before surgery, the chances of graft survival and the risk of relapse, donor sufficiency, the number of sessions required for a good cosmetic effect, wound healing time, and the risk of complications such as hypertrophic scars, infection, and corkscrew hair [5]. Additionally, three studies describe the development of LPP as a complication in 28 patients who underwent HT to treat other types of alopecia [6,7,8]. A systematic review identifying articles published between 1960 and 2019 revealed only fifteen reports describing 34 patients with primary scarring alopecias, including only five case reports describing 5 patients with LPP [9]. Two years later, other authors published a systematic review reporting eight patients with LPP treated with HT [10]. Properly designed and controlled clinical trials of HT in individuals with LPP, required to formulate definitive, evidence-based recommendations concerning this subject, are lacking. A series of case studies will not answer these questions, but given the scarcity of better-quality evidence, even such data are an important contribution to the collection of scientific evidence, which is important in planning clinical trials.

The authors of both systematic reviews highlighted the problem of publication bias, which can overestimate treatment effects based on literature analysis. Thus, the purpose of our paper is to present a series of five consecutive patients with LPP, regardless of outcome.

## 2. Case Presentation

All patients eligible for surgery had a confirmed diagnosis of LPP. This diagnosis was established either at our clinic or confirmed by our team if initially made elsewhere. Confirmation involved both trichoscopic and HP examination. Trichoscopic criteria indicative of LPP included the following: smooth, shiny, hairless patches; white cicatricial areas; fibrotic white dots; white areas of fibrosis; and the absence of erythema, red or yellow dots, scales, visible follicular openings, and hyperkeratosis. Histopathological diagnosis required the confirmed presence of a fibrosing band of fibroplasia in a scalp biopsy. At the time of qualification, patients were required to have a history of past LPP symptoms but could not exhibit active disease.

A thorough medical history was obtained from each patient before the procedure, including information on medications, chronic diseases, allergies, and continuously taken medications. The following laboratory tests were ordered and evaluated: morphology, hepatitis B, hepatitis C, HIV, coagulation system, AST, ALT, urea, and creatinine. The scalp was evaluated using trichoscopy (at 10× and 70× magnification). Patients qualified for the procedure were not treated with glucocorticosteroids immediately prior to transplantation (they had to be in remission for at least 6 months). Transplantation was planned from the occipital region to the front of the head, including the corners, middle of the head, and top of the head. Hairline correction was planned according to individual needs. The frontal line was drawn and measured, and the course of the line was discussed with the patient, who accepted it. Platelet-rich plasma was collected directly prior to the procedure and administered instantly as mesotherapy to increase graft survival and accelerate healing. The procedure was performed under sterile conditions. The recipient area was contoured after disinfecting the scalp three times with Kodan and washing with NaCl 0.9%. The occipital region was topically anesthetized with a mixture consisting of 250 mL NaCl 0.9% (Polpharma, Starogard Gdański, Poland), 20 mL lignocaine 2% (Polfa Warszawa, Warsaw, Poland), 20 mL bupivacainum hydrochloricum 0.5% (Polfa Warszawa, Warsaw, Poland), 2 mL natrium bicarbonicum 8.4% (Polpharma, Starogard Gdański, Poland), and 1 mL adrenaline 0.1% (Polpharma, Starogard Gdański, Poland). After 20 min, the grafts were retrieved using the FUE method with a 0.95 drill. The retrieved grafts were evaluated under a microscope, and a sterile dressing was applied to the occipital region. The corners and front of the head were anesthetized using the same method as described above. A WAW FUE 1.2 hybrid blade was used to create holes in the front of the head, middle, and tip. Platelet-rich plasma (PRP) was administered to ensure better survival of follicular units [9], and gentian was used to make the previously created holes visible. PRP isolation was performed as follows: 10 mL of peripheral blood was collected into a certified tube (RegenLab, Le Mont-sur-Lausanne, Switzerland) and centrifuged using an iFuge D06 centrifuge (Gujarat, India) for 8 min at 2000 RPM. The resulting PRP was then administered via microneedle mesotherapy to both the recipient and donor areas.

The previously harvested grafts were placed in the created holes. No excessive bleeding was observed during or after implantation. The symmetry and placement of the grafts and the anterior line were checked. After the procedure, the scalp was irradiated with a Tri-Wave LED lamp (Dermalux, Warrington, UK) to accelerate healing. Vital parameters (blood pressure, heart rate, saturation) were monitored throughout the procedure, and they remained within the normal range. Patients were discharged in good general and local conditions, with written recommendations that included not driving and contacting the clinic in case of complications.

**Figure 1:**
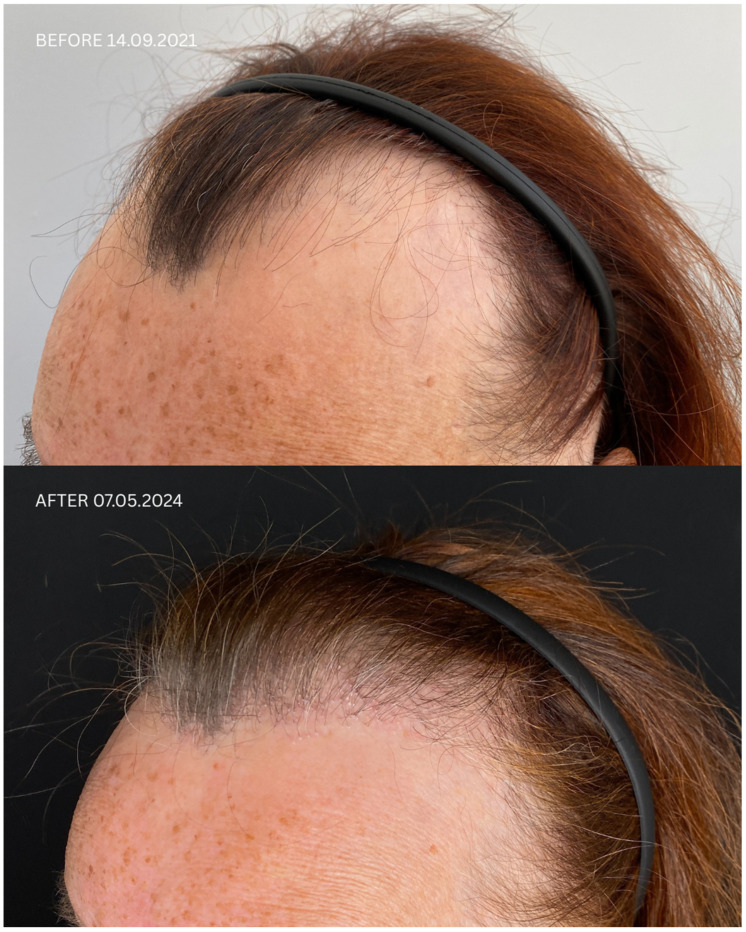

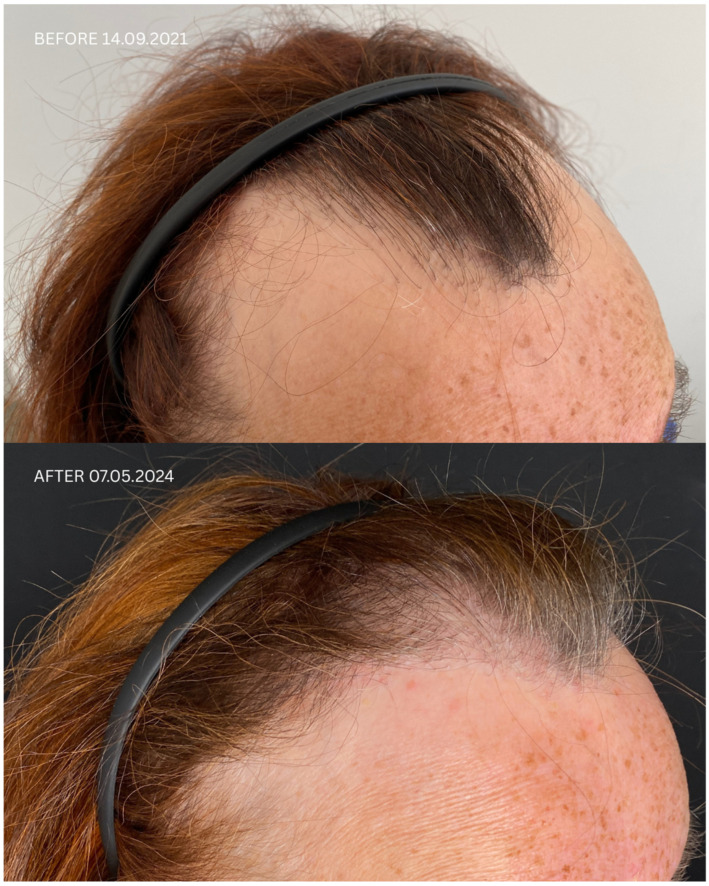

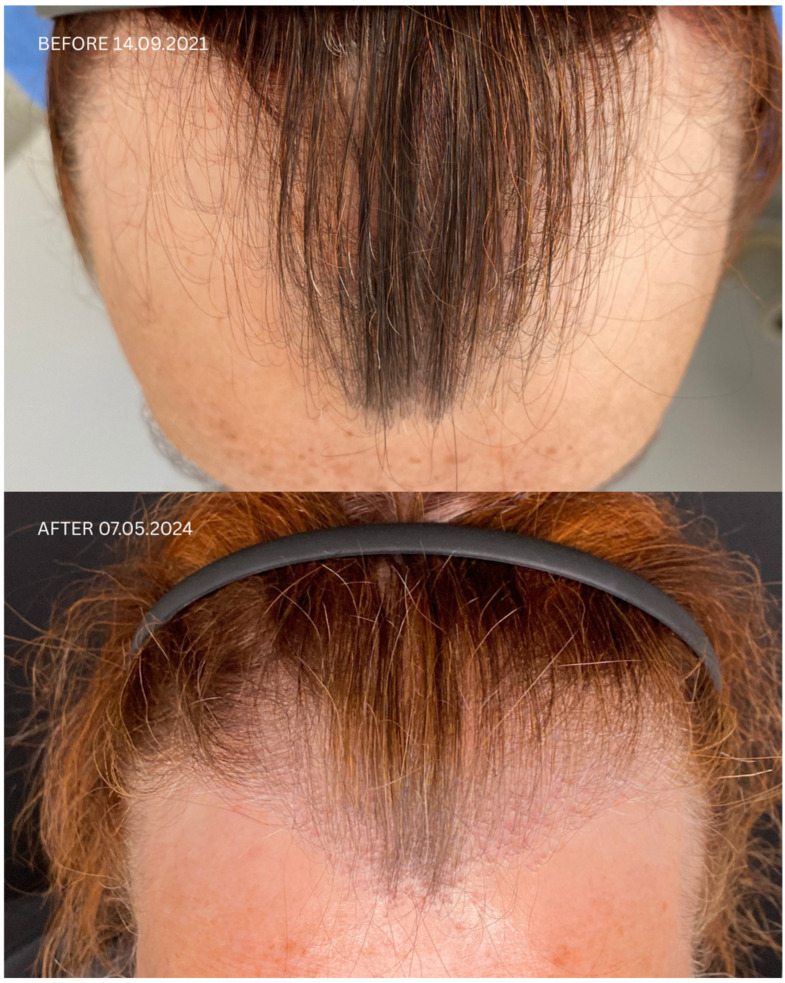
Comparison of Patient 1’s lateral and frontal views before (upper panels) and after (lower panels) hair transplantation.

**Figure 2:**
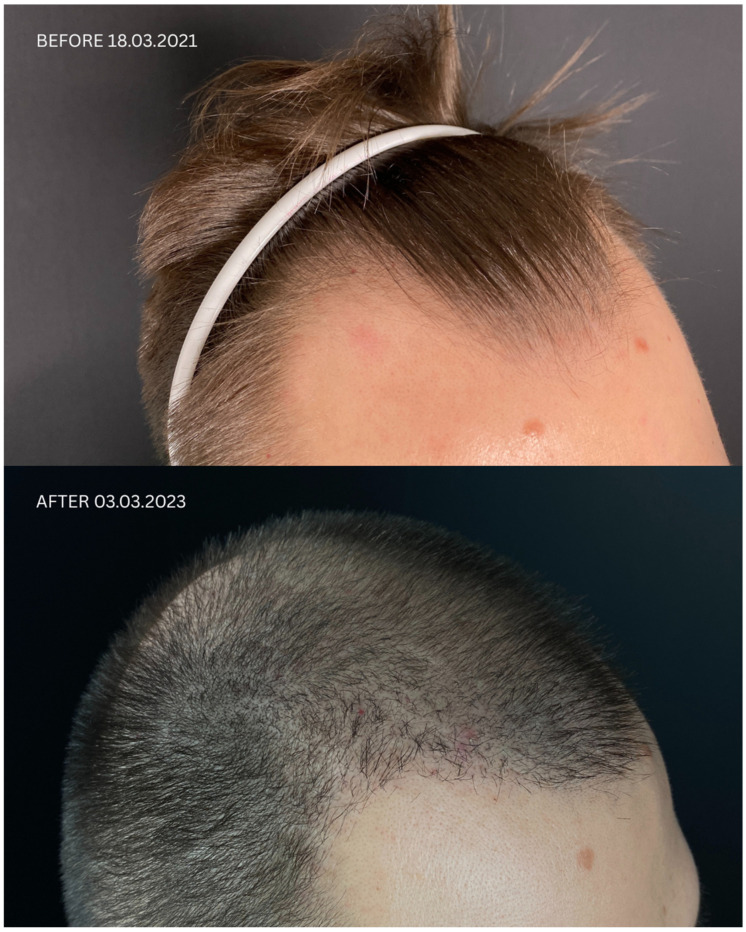

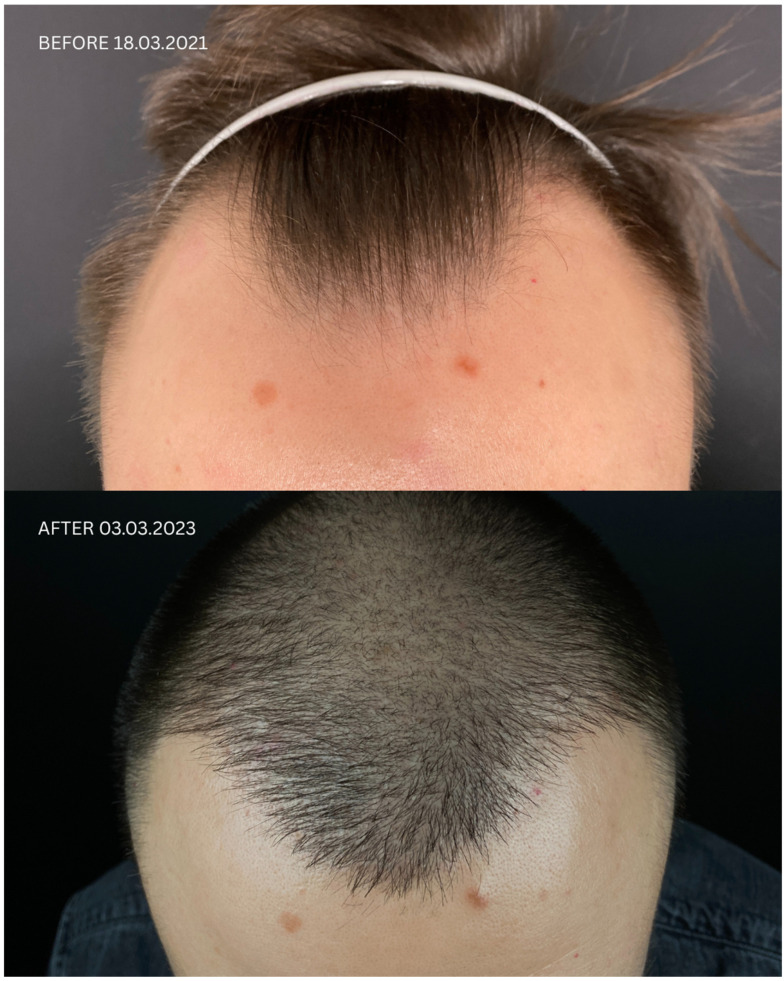

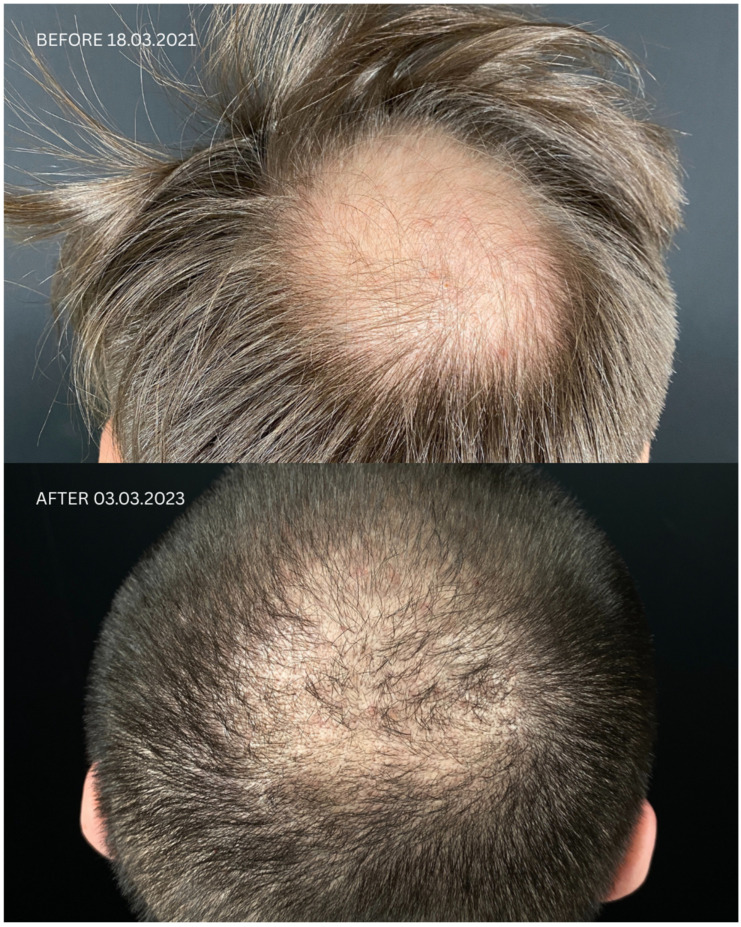
Comparison of Patient 2’s lateral, frontal, and posterior views before (upper panels) and after (lower panels) hair transplantation.

**Figure 3:**
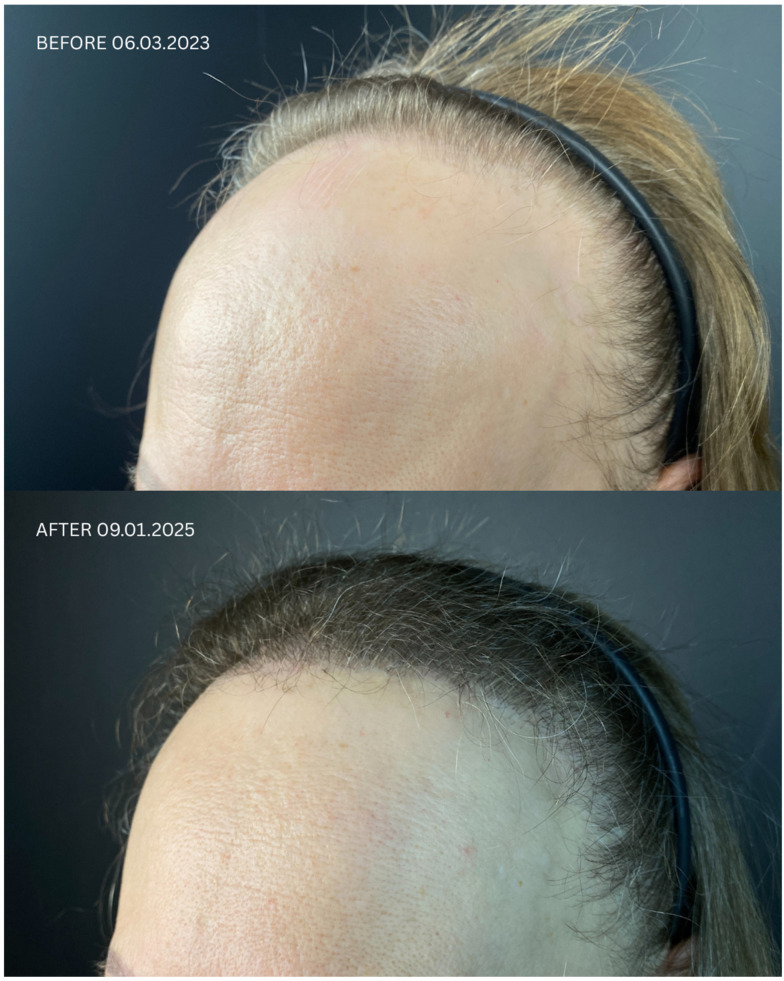

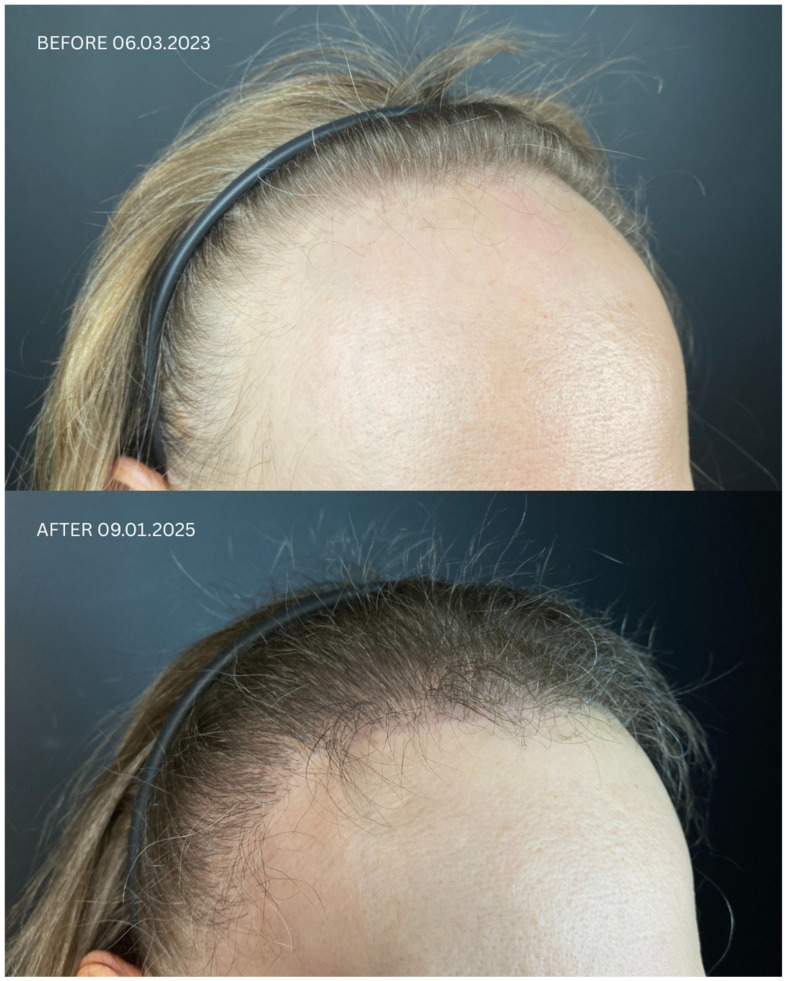

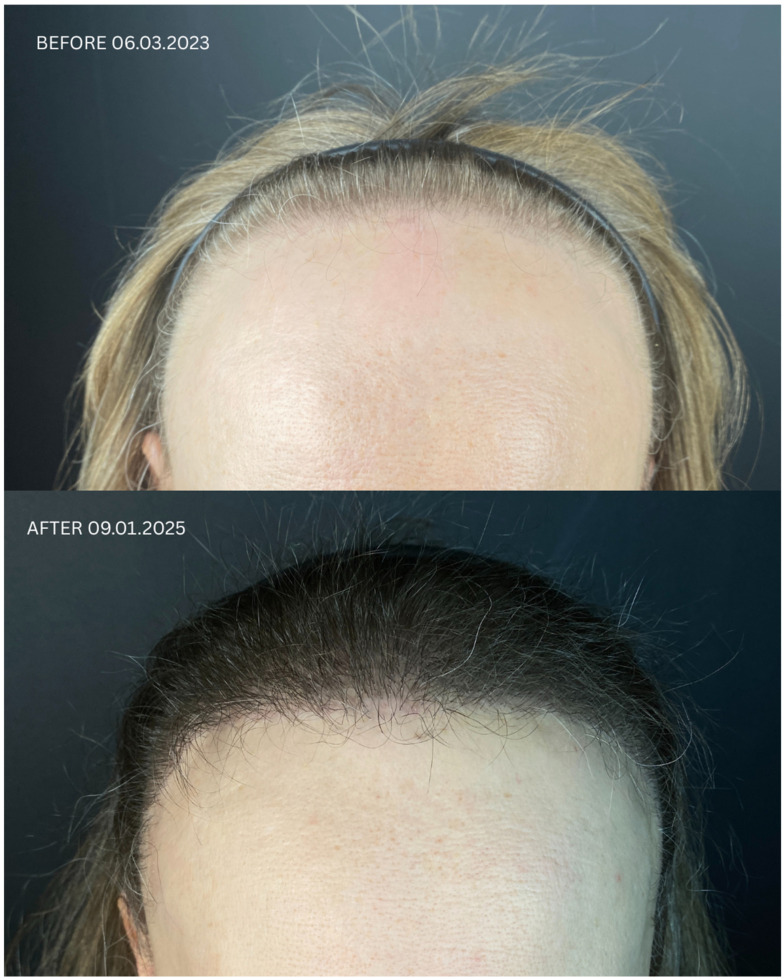
Comparison of Patient 3’s lateral and frontal views before (upper panels) and after (lower panels) hair transplantation.

**Figure 4:**
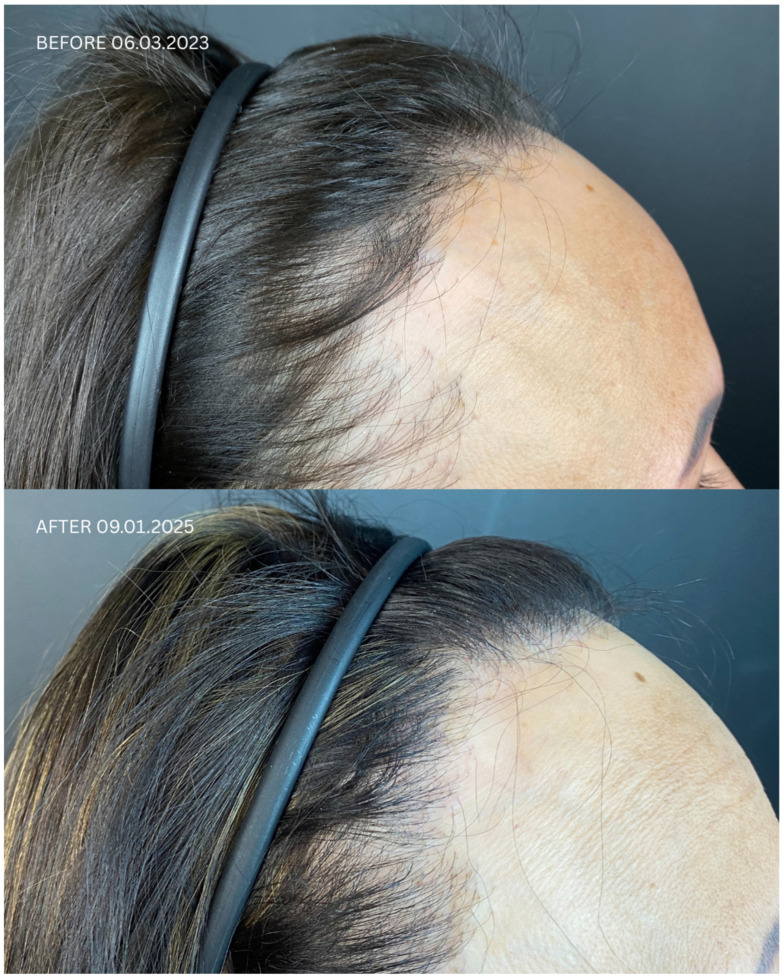

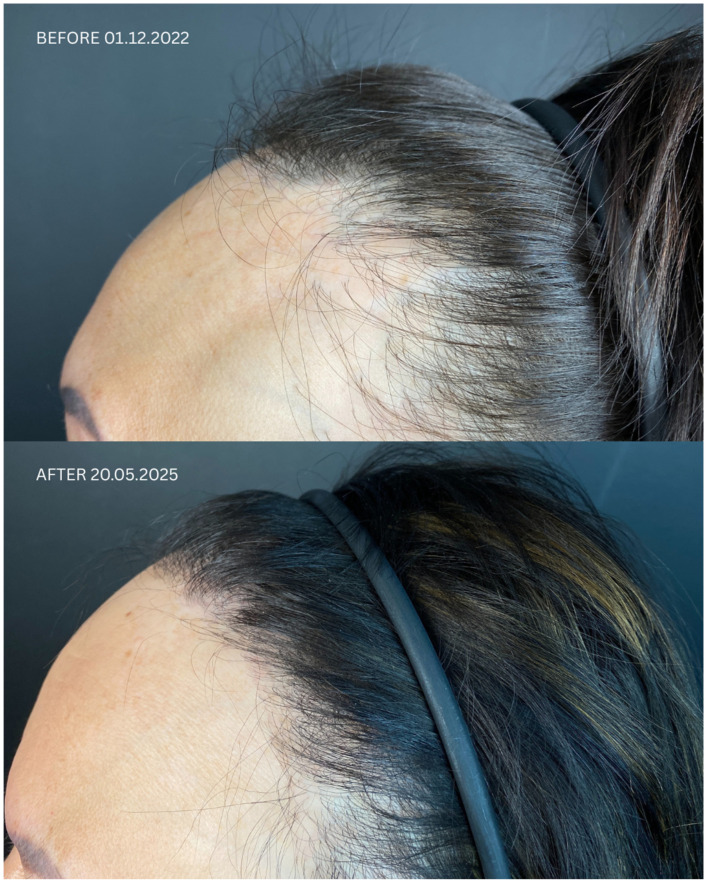

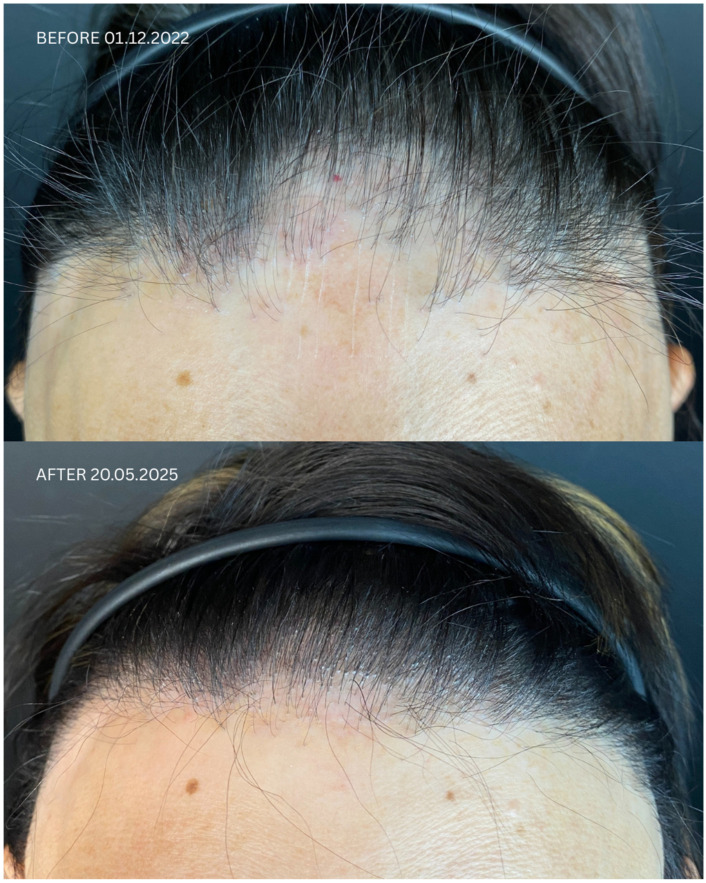
Comparison of Patient 5’s lateral and frontal views before (upper panels) and after (lower panels) hair transplantation.

Detailed information regarding all patients is presented in Table 1 and Table 2, and in Figure 1, Figure 2, Figure 3 and Figure 4. Briefly, initial satisfaction with the treatment varied (ranging from 1 to 5), but increased in all patients one year after the procedure. Patients who were initially dissatisfied explained the change in their rating by the gradual improvement in cosmetic outcomes over time. No adverse events were reported in any of the patients during long-term follow-up.

## 3. Discussion and Conclusions

According to common belief, hair loss in LPP is irreversible because hair regrowth in scarring alopecia is not possible due to the autoimmunological basis of the disease. The precise cause of LPP remains unclear; however, it is believed that T-lymphocytes erroneously target follicular antigens, leading to the selective destruction of hair follicles [12]. In recent years, there has been growing interest in the use of biological agents—such as TNF-α inhibitors, IL-17 inhibitors, IL-23 inhibitors, JAK inhibitors, and IFNAR1 inhibitors—for the treatment of LPP, i.e., outside of their approved indications [13]. A single case report showed complete hair regrowth in a patient with histopathologically confirmed LPP treated with ixekizumab—an anti-psoriatic, anti-interleukin (IL)-17A/F, humanized IgG4 monoclonal antibody [14]; this drug had previously proven effective in inhibiting the active phase of the disease [15]. A case report even documented partial hair regrowth in a patient with dyskeratosis congenita after three months of treatment with adalimumab [16] and in one patient with LPP [17]. There is also a reported case of a patient with central centrifugal cicatricial alopecia who experienced partial hair regrowth in the medial parietal scalp after a two-month trial of baricitinib [18]. The visual cosmetic effect of this treatment was variable—better than HT in some cases, worse in others. Due to the lack of standardized endpoints, these results cannot be directly compared.

Systematic reviews [9,10,19] indicated an approximate 75% rate of favorable outcomes of HT among patients with LPP. In our case series, we observed a similar 60% (3/5) success rate in the near follow-up and even better in the extended follow-up. The increased satisfaction in the long-term follow-up was attributed by patients to the improvement in cosmetic outcome over time. This observation is consistent with the literature, which indicates that the final effect is typically achieved after at least 12 months [20]. Our case series is not powered to confirm this success rate due to subjective nature of outcomes and the limited sample size. It is also too small to assess the impact of patient characteristics on clinical response; therefore, we can only report our findings with a very cautious interpretation. However, this paper provides a premise supporting such estimates and contributes to the growing body of case reports for the future.

Key factors influencing patient satisfaction with LPP after FUE are presented in Table 3. In a cohort of this size and with the type of data we collected, we did not observe any demographic, clinical, or therapeutic factors accounting for differences in patient satisfaction. Paradoxically, patient no. 4, who remained in remission the longest—presumably a positive predictor of treatment efficacy and, consequently, satisfaction—reported the lowest possible level of satisfaction. Our team’s subjective assessment of cosmetic outcomes, which unanimously indicated no significant differences in therapeutic response between patients and no correlation between patients’ dissatisfaction with cosmetic results and the physicians’ evaluation, points to challenges in defining and assessing endpoints. Although numerous patient-reported outcome measures (PROMs) exist for alopecia, they are inadequate. Even in the more prevalent alopecia areata, for which a broader range of scales is available than in LPP, the existing tools have proven insufficient [21]. The highly simplified scale we used may have led patients to skip over detailed questions, preventing them from reflecting on or recognizing multiple aspects of change. The non-validated and entirely subjective method of assessment may also be sensitive to factors such as the patient’s mental state. It has previously been noted in the medical law literature that hair transplantation, as an elective procedure, is associated with higher patient expectations and lower tolerance for the inevitable consequences of surgical intervention, such as pain or swelling, which in the early post-operative period may negatively affect patient satisfaction [22]. According to the cited authors, particular attention should be given to photographic documentation, which should be conducted in a way that enables maximally precise, quantitative, and reproducible assessment of changes in hair density. Furthermore, the authors highlighted the importance of assessing patients’ mental health status. In our study, we did not assess patients’ baseline mental health, as this is a sensitive issue in the context of commercial elective procedures. Moreover, based on both the literature and clinical experience, it is well known that LPP negatively impacts patients’ psychological well-being, and we anticipated low mood among all included patients. However, the existing literature indicates that certain personality traits are associated with dissatisfaction with treatment. Problematic cosmetic patient characteristics include unrealistic expectations, unhappy tendencies, body dysmorphic disorder, over-flattering behavior, perfectionism, poor self-image, cyberchondriac behavior, history of trauma, VIP status, poor compliance, expectation of immediate results, and general pessimism [23]. Moreover, and perhaps less intuitively, factors such as income level, educational attainment, motivation for undergoing transplantation, and early life experiences significantly affect satisfaction following hair transplantation [24]. In the same study, no association was found between satisfaction and gender, age, marital status, or family history of alopecia [24]. Unfortunately, as we were unaware of this publication at the time of data collection, these variables were not controlled for in our sample. It is possible that expanding the medical history to include psychiatric comorbidities and implementing a quantitative, validated preoperative mood assessment could have helped identify patients at elevated risk of dissatisfaction. Such patients might benefit from additional preoperative counseling and more intensive post-operative monitoring, potentially improving early satisfaction with treatment outcomes.

Moreover, a critical component of our procedure was the administration of PRP before the intervention. PRP is utilized in the treatment of various hair and skin conditions. Recent research has explored its effectiveness in managing both scarring (cicatricial) and non-scarring (noncicatricial) forms of hair loss. PRP contains alpha granules that release a range of bioactive proteins, including platelet-derived growth factor, transforming growth factor, vascular endothelial growth factor, insulin-like growth factor, epidermal growth factor, and interleukin-1 [25]. Its standalone administration proved effective in androgenetic alopecia and inconclusive in alopecia areata; in scarring alopecia, the efficacy of PRP administered alone in patients with LPP appeared promising [26], but the number of reports describing the combination of PRP with FUE available in the literature is very limited [27,28,29]. There have been reports of LPP occurring in a total of 28 patients who underwent hair transplantation or facelift procedures [7,8,30], although none of the individuals had a prior history of LPP or FFA. The reason for this observation is the Koebner phenomenon, i.e., the occurrence of LP, psoriasis, or vitiligo, as a complication triggered by surgery or skin trauma. One suggested mechanism behind the Koebner phenomenon involves an initial non-specific inflammatory response to skin injury, followed by a more targeted, disease-specific immune reaction involving T-cells, B-cells, or the infiltration of autoantibodies [11]. Koebnerization develops in only 5.9% of patients from the general population following HT [8], while these rates are much higher (28% and 38%) in adults and children with LP, respectively [31]. Some genes may be predictors of the risk of this phenomenon in patients with LPP, but data are too scarce for clinical use at the current stage [32]. KP is a sign of reactivation of an underlying disease; therefore, it is treated with the same drugs as the primary lesions. In our case series, one patient exhibited early signs of desquamation and was advised to use topical corticosteroids every other day for two weeks, which resolved the problem.

Perspectives for hair transplantation in LPP are unclear. Large controlled studies are required to confirm the effectiveness of biological drugs for hair regrowth and to compare their efficacy with HT. Subsequently, it could be considered to supplement the FUE+PRP protocol with biological drugs or stem cells, which have improved the effectiveness of hair transplantation in scars of non-LPP origin; however, there is currently no literature that allows the efficacy of such an approach to be estimated in patients with LPP [33,34]. In summary, although traditionally considered irreversible, hair loss in LPP may, under specific conditions, partially respond to immunomodulatory treatment or hair transplantation combined with regenerative approaches such as PRP. While our findings support the feasibility and cosmetic benefit of HT+PRP in selected cases, large-scale prospective studies are needed to establish standardized criteria for treatment selection and to compare these strategies with biological therapies in terms of long-term efficacy.

## Figures and Tables

**Table 1:** Summary of clinical characteristics of patients and their therapies.

Patient ID	Sex	Date of Diagnosis	Diagnostic Basis	Lesions in a Location Other Than the Scalp		Number of Months in Remission Before HT	Treatment in 14-Day Post-Operative Phase	Permanent Treatment After HT	Remission Duration Since the Day of HT	Date of HT	Number of Grafts	Post-Operative Procedures	Number of Follow-Up Visits from HT to Data Cut-Off Date	FU Visit, 12 Months After HT	Follow-Up in 2025
				Before HT	After HT										
1	F	FFA 21 January 2021	HP+ TS+	No	No	8 months	Solacutan Pirolam Clobex Finahit Minoxidil 1.25 mg Phototherapy (UV) once daily	Minoxidili 1.25 mg Lactosi gs M.F. pulvis D.t.d. No. 30 PRP	26 months Second procedure on 18 October 2022	14 September 2021	1700 + 2900 + 3200	Tri-Wave MD Lamp, red light (633 nm, 20 min), + mesotherapy with PRP: 3 administrations every 30 days, the 4th administration 6 months after transplantation	4	Remission of LLP, no recurrence of LLP disease on clinical and trichoscopic examination Maintenance treatment: minoxidili 1.25 mg Lactosi gs M.F. pulvis D.t.d. No. 40, Finaster/Adadut	Remission of LLP, no recurrence of LLP disease on clinical and trichoscopic examination
2	M	23 October 2020	HP+ TS+	Close to the scar	No	5 months	Finahit Metronidasol Phototherapy (UV)	Finapil Curacn Alopexy PRP	32 months (second procedure on 11 February 2022)	18 March 2021	2500 + 2900	Tri-Wave MD Lamp, red light (633 nm, 20 min) + mesotherapy with PRP: 3 administrations every 30 days, the 4th administration 6 months after transplantation	4	Remission of LLP, no recurrence of LLP disease on clinical and trichoscopic examination Maintenance treatment: Minoxidili 0.125 mg Lactosi gs M.F. pulvis D.t.d. No. 30, Finapil	Treatment finished
3	F	16 November 2019	HP+ TS+	No	No	6 months		Finasteridum Bluefish, Spironol, Minoxidil, Alpicort E, PRP		6 March 2023	1500	Tri-Wave MD Lamp, red light (633 nm, 20 min) + mesotherapy with PRP: 3 administrations every 30 days, the 4th administration 6 months after transplantation	1		
4	F	FFA 16 November 2019	HP+ TS+	No	No	40 months	Finasteride Bluefish Minoksidili 0.125 mg Phototherapy (UV)	Finasteridum Bluefish, Minoksidil 0.125 mg PRP	19 months	6 March 2023	1500	Tri-Wave MD Lamp, red light (633 nm, 20 min) + mesotherapy with PRP: 3 administrations every 30 days, the 4th administration 6 months after transplantation	2	Remission of LLP, no recurrence of LLP disease on clinical and trichoscopic examination Maintenance treatment: Minoxidili 0.00125 Lactosi gs M.F. pulvis D.t.d. No. 40, Finaster	Remission of LLP, no recurrence of LLP disease on clinical and trichoscopic examination
5	F	FFA 23. June 2021	HP+ TS+	No	No	6 months	Axotret Dermovate Dostinex Finasteride Stada Phototherapy (UV)	Axotret PRP	3 months (second procedure on 24 July 2024)	1 December 2022	1655	Tri-Wave MD Lamp, red light (633 nm, 20 min) + mesotherapy with PRP: 3 administrations every 30 days, the 4th administration 6 months after transplantation	3	Remission of LLP, no recurrence of LLP disease on clinical and trichoscopic examination Maintenance treatment: Minoxidili 1.25 mg Lactosi gs M.F. pulvis D.t.d. No. 40, Spironol	Remission of LLP, no recurrence of LLP disease on clinical and trichoscopic examination

D.t.d. No. 40—dentur tales doses numero 40 (the prescription is for 40 individual doses), F—female, FFA—frontal fibrosing alopecia, FU—follow-up, HP(+), lesions typical of LPP on histopathological examination [11]; HT—hair transplantation, M—male, min—minutes, M.F.—misce fiat (mix to make a powder), nm—nanometers, no—number, PRP—platelet-rich plasma, qs—quantum satis (as much as needed), TS (+), lesions typical of LPP on trichoscopic examination [11]; UV—ultraviolet.

**Table 2:** Patient’s baseline characteristics and early satisfaction from treatment.

Patient	Gender	Age	Time from Onset	Patient Satisfaction	
				Early	After 12 Months
Patient 1	female	75	8 years	4	4–5
Patient 2	male	33	10 years	3	4–5
Patient 3	female	56	Since adolescence	1	4–5
Patient 4	female	46	2 years	1	4–5
Patient 5	female	43	2 years	5	5

**Table 3:** Critical points for patient satisfaction after hair transplantation.

Stage	Suggestion
Before surgery	**Dermatoscopic evaluation** to determine the cause of hair loss.
	**Donor area assessment** Determine hair density, quality, and the number of follicular units that can be safely harvested without significantly worsening the donor area’s appearance.
	**Recipient area assessment:** Thoroughly check that the recipient area is completely free of LPP signs, even at a subclinical level. LPP can also affect the donor area, which would lead to damage or loss of extracted grafts after transplantation.
	**Laboratory tests and confirmation of stable remission:** Hair transplantation in active LPP is contraindicated. In that case, the transplanted hair follicles will be destroyed by the ongoing inflammatory process, and the procedure itself may even trigger disease reactivation.
	**Managing patient expectations:** Clearly communicate to the patient that the graft survival rate in scarred areas may be lower than in standard transplants, and density may be limited. Multiple procedures are often needed to achieve a satisfactory result. Explain that the cosmetic outcome may be inferior to transplants into healthy skin, and that initial effects will take at least 6 months, with the full effect expected no sooner than 12 months.
	**Individualized hairline design** tailored to facial features and age
	**Planning immunosuppressive treatment** before, during, and after surgery.
During surgery	**Recommended technique is Follicular Unit Extraction** as it is less invasive and carries a lower risk of additional scarring in the donor area.
	**Platelet-Rich Plasma (PRP):** PRP application may enhance procedure effectiveness by improving graft survival and scar tissue vascularization.
	**Precise micro-incisions** in the scarred areas are crucial for minimizing trauma and optimizing blood supply
	**Lower graft density in scarred areas:** Due to poorer blood supply, grafts should be implanted at a lower density in scarred areas than in standard transplants (e.g., 30–40 units/cm^2^ instead of 50–70 units/cm^2^) to prevent competition among follicles for limited blood supply and to avoid graft necrosis.
After surgery	**Ongoing dermatological follow-up:** The patient must remain under constant dermatological care to monitor for potential LPP reactivation and implement appropriate treatment.
	**For scientific studies:** Evaluations at 6 and 12 months are too infrequent to monitor effects with the sensitivity needed to capture inter-individual differences required for drawing scientific conclusions. For research purposes, follow-up visits should be conducted more frequently.

## Data Availability

Additional data are available upon reasonable request, after anonymization.

## References

[1] Naeini F.F., Saber M., Faghihi G. (2021). Lichen planopilaris: A review of evaluation methods. Indian J. Dermatol. Venereol. Leprol..

[2] Cardoso C.O., Tolentino S., Gratieri T., Cunha-Filho M., Lopez R.F., Gelfuso G.M. (2021). Topical treatment for scarring and Non-Scarring alopecia: An overview of the current evidence. Clin. Cosmet. Investig. Dermatol..

[3] Aukerman E.L., Jafferany M. (2023). The psychological consequences of androgenetic alopecia: A systematic review. J. Cosmet. Dermatol..

[4] Ujiie H., Rosmarin D., Schön M.P., Ständer S., Boch K., Metz M., Maurer M., Thaci D., Schmidt E., Cole C. (2022). Unmet Medical Needs in Chronic, Non-communicable Inflammatory Skin Diseases. Front. Med..

[5] Singh S., Muthuvel K. (2021). Role of Hair Transplantation in Scarring Alopecia-To Do or Not to Do. Indian J. Plast. Surg..

[6] Crisóstomo M.R., Crisóstomo M.G.R., Crisóstomo M.C.C., Gondim V.J.T., Benevides A.N. (2011). Hair loss due to lichen planopilaris after hair transplantation: A report of two cases and a literature review. An. Bras. Dermatol..

[7] Donovan J. (2012). Lichen planopilaris after hair transplantation: Report of 17 cases. Dermatol. Surg..

[8] Chiang Y.Z., Tosti A., Chaudhry I.H., Lyne L., Farjo B., Farjo N., Cadore de Farias D., Griffiths C., Paus R., Harries M. (2012). Lichen planopilaris following hair transplantation and face-lift surgery. Br. J. Dermatol..

[9] Ekelem C., Pham C., Atanaskova Mesinkovska N. (2019). A Systematic Review of the Outcome of Hair Transplantation in Primary Scarring Alopecia. Skin. Appendage Disord..

[10] Lee J.A., Levy D.A., Patel K.G., Brennan E., Oyer S.L. (2021). Hair Transplantation in Frontal Fibrosing Alopecia and Lichen Planopilaris: A Systematic Review. Laryngoscope.

[11] Ueki H. (2005). Koebner phenomenon in lupus erythematosus with special consideration of clinical findings. Autoimmun. Rev..

[12] Errichetti E., Figini M., Croatto M., Stinco G. (2018). Therapeutic management of classic lichen planopilaris: A systematic review. Clin. Cosmet. Investig. Dermatol..

[13] Agarwal P., Ajuchi O., Lukowiak T.M., Rao B.K. (2025). Off-label use of biologics and janus kinase (JAK) inhibitors for scarring alopecias: A narrative review. Arch. Dermatol. Res..

[14] Ahmed H., Petkar M., Steinhoff M. (2023). Successful treatment of rare linear lichen planopilaris with Ixekizumab. J. Dermatolog. Treat..

[15] Peters G., Pina K., Posligua A., Durkin J. (2024). 53358 Ixekizumab in Patients with Lichen Planopilaris—An Open-Label Study. J. Am. Acad. Dermatol..

[16] Takahashi T., Yamasaki K., Terui H., Omori R., Tsuchiyama K., Fujimura T., Aiba S. (2019). Perifolliculitis capitis abscedens et suffodiens treatment with tumor necrosis factor inhibitors: A case report and review of published cases. J. Dermatol..

[17] Alam M.S., LaBelle B. (2020). Treatment of lichen planopilaris with adalimumab in a patient with hidradenitis suppurativa and rheumatoid arthritis. JAAD Case Rep..

[18] Workman K., Kindred C. (2023). Hair regrowth in a patient with central centrifugal cicatricial alopecia after a 2-month trial of baricitinib. JAAD Case Rep..

[19] Kumar A.R., Ishii L.E. (2020). Hair transplantation for scarring alopecia. Facial Plast. Surg. Clin. N. Am..

[20] Avram M.R., Finney R., Rogers N. (2017). Hair transplantation controversies. Derm. Surg..

[21] Darchini-Maragheh E., Moussa A., Yoong N., Bokhari N., Jones L., Sinclair R. (2025). Alopecia Areata–Specific Patient-Reported Outcome Measures: A Systematic Review. JAMA Dermatol..

[22] Sattur S.S., Sattur S.I. (2021). Patient Counselling and Medicolegal Aspects of Hair Transplant Surgery. Indian J. Plast. Surg..

[23] Desai V. (2021). Managing an Unhappy Patient. Indian J. Plast. Surg..

[24] Liu Y., Liu F., Qu Q., Fan Z.-X., Miao Y., Hu Z.-Q. (2019). Evaluating the Satisfaction of Patients Undergoing Hair Transplantation Surgery Using the FACE-Q Scales. Aesth. Plast. Surg..

[25] Paichitrojjana A., Paichitrojjana A. (2022). Platelet Rich Plasma and Its Use in Hair Regrowth: A Review. Drug Des. Devel Ther..

[26] Behrangi E., Akbarzadehpasha A., Dehghani A., Zare S., Ghassemi M., Zeinali R., Goodarzi A., Lotfi Z. (2024). Platelet-rich plasma as a new and successful treatment for lichen planopilaris: A controlled blinded randomized clinical trial. J. Cosmet. Dermatol..

[27] Saxena K., Saxena D.K., Savant S.S. (2016). Successful Hair Transplant Outcome in Cicatricial Lichen Planus of the Scalp by Combining Scalp and Beard Hair Along With Platelet Rich Plasma. J. Cut. Aesth. Surg..

[28] Garg S., Pandya I., Bhatt S. (2019). Follicular Unit Extraction (FUE) Hair Transplantation in Combination with Platelet Rich Plasma for the Treatment of Scarring Alopecia: A Case Series. Arch. Clin. Med. Case Rep..

[29] Garg S., Manchanda S. (2017). Platelet-rich plasma-an ‘Elixir’ for treatment of alopecia: Personal experience on 117 patients with review of literature. Stem Cell Investig..

[30] Kossard S., Shiell R.C. (2005). Frontal fibrosing alopecia developing after hair transplantation for androgenetic alopecia. Int. J. Dermatol..

[31] Zhang X., Lei L., Jiang L., Fu C., Huang J., Hu Y., Zhu L., Zhang F., Chen J., Zeng Q. (2023). Characteristics and pathogenesis of Koebner phenomenon. Exp. Dermatol..

[32] Shan D., Long H., Lai W. (2017). TRPA1 may contribute to the exacerbation of oral lichen planus through Koebner phenomenon. Oral. Dis..

[33] Popescu F.M., Filip L., Popescu M., Florescu I.P. (2024). Stem cell therapy prior to follicular unit hair transplantation on scarred tissue: A novel approach to a successful procedure. J. Med. Life.

[34] Rajan A., Rudnicka L., Szepietowski J.C., Lallas A., Rokni G.R., Grabbe S., Goldust M. (2022). Differentiation of frontal fibrosing alopecia and Lichen planopilaris on trichoscopy: A comprehensive review. J. Cosmet. Dermatol..

